# Genes and Eating Preferences, Their Roles in Personalized Nutrition

**DOI:** 10.3390/genes11040357

**Published:** 2020-03-27

**Authors:** Anna Vesnina, Alexander Prosekov, Oksana Kozlova, Victor Atuchin

**Affiliations:** 1 Department of Bionanotechnology, Kemerovo State University, 650043 Kemerovo, Russia; koledockop1@mail.ru (A.V.); ms.okvk@mail.ru (O.K.); 2 Laboratory of Biocatalysis, Kemerovo State University, 650043 Kemerovo, Russia; aprosekov@rambler.ru; 3 Laboratory of Optical Materials and Structures, Institute of Semiconductor Physics, 630090 Novosibirsk, Russia; 4 Laboratory of Semiconductor and Dielectric Materials, Novosibirsk State University, 630090 Novosibirsk, Russia; 5 Research and Development Department, Kemerovo State University, 650000 Kemerovo, Russia

**Keywords:** nutrigenetics, eating preferences, genotype, polymorphism, functional product, personalized nutrition

## Abstract

At present, personalized diets, which take into account consumer genetic characteristics, are growing popular. Nutrigenetics studies the effect of gene variations on metabolism and nutrigenomics, which branches off further and investigates how nutrients and food compounds affect genes. This work deals with the mutations affecting the assimilation of metabolites, contributing to nutrigenetic studies. We searched for the genes responsible for eating preferences which allow for the tailoring of personalized diets. Presently, genetic nutrition is growing in demand, as it contributes to the prevention and/or rehabilitation of non-communicable diseases, both monogenic and polygenic. In this work, we showed single-nucleotide polymorphisms in genes—missense mutations that change the functions of coded proteins, resulting in a particular eating preferences or a disease. We studied the genes influencing food preferences—particularly those responsible for fats and carbohydrates absorption, food intolerance, metabolism of vitamins, taste sensations, oxidation of xenobiotics, eating preferences and food addiction. As a result, 34 genes were identified that affect eating preferences. Significant shortcomings were found in the methods/programs for developing personalized diets that are used today, and the weaknesses were revealed in the development of nutrigenetics (inconsistency of data on SNP genes, ignoring population genetics data, difficult information to understand consumer, etc.). Taking into account all the shortcomings, an approximate model was proposed in the review for selecting an appropriate personalized diet. In the future, it is planned to develop the proposed model for the compilation of individual diets.

## 1. Introduction

To date, the personalized approach to the person, as based on the 4P principles (personalized, predictive, preventative, participative), both in the fields of medicine and nutrition, plays an important role, because the prevention and treatment of the body depend on the right rational nutrition [1]. Proper nutrition is part of a healthy lifestyle, and its disturbances cause various diseases, both monogenic (depending on a mutation in one single gene, e.g., phenylketonuria, lactose intolerance, celiac disease) and polygenic (depending on the changes in a number of genes, as well as environmental factors, e.g., cancer, diabetes, etc.). Therefore, an individual approach to the nutrition based on genetics becomes crucial.

The study has the following aims:

1) Searching for the scientific data about the genes, in which oligonucleotide polymorphisms affect metabolites absorption and overall well-being.
2) Searching for the information on the achievements in nutrigenetics contributing to the development of personalized diets.

In the near future, we plan to model personalized diets and develop technologies for the production of functional products which play an important role in nutrition.

## 2. Genes Responsible for Eating Preferences

Nutrigenetics studies the genetic basis of why people react differently to identical nutrients. The research in this field creates the basis for the development of personalized nutrition, aimed at solving individual health problems, because a person’s health, immune system response and psycho-emotional state largely depend on metabolism. The individual diets are based on the genetic information analysis for which a list of genes is required.

As part of the systematic review, genes and their polymorphisms were searched. Three reviewers were involved in the literature search. The review was carried out in: National Center for Biotechnological Information (NCBI), i.e., in PubMed and LitVar, GeneCards, SNPedia, Google Scholar, Web of Science, 1000 genomes (1KGP), Russian Scientific Electronic Library (https://www.elibrary.ru) and https://cyberleninka.ru. The search was implemented for genes and their polymorphisms, diseases and eating preferences. The upper limit of the date was 09 December 2019 and the lower limit was not set. It was carried out in Russian and English, focused on research conducted with people, and included only free full-text articles in the public domain. As a result, 86 articles were included in the review. We found the following genes and divided them into seven categories depending on their affect.

### 2.1. Genes Responsible for the Digestion and Absorption of Carbohydrates and Fats

There are nine genes responsible for the absorption of carbohydrates and fats: ADRB2 (rs1042714 and rs1042713polymorphisms), TCF7L2 (rs12255372, rs7903146), FABP2 (rs1799883), PPARG (rs1801282), CETP (rs5882), ADRB3 (rs4994), A5 (s662799, rs3135506), LEPR (rs1137101), ApoE(rs429358, rs7412). Besides, we analyzed the dynamics of genetic composition of populations, namely frequencies of dominant and recessive alleles (Table 1).

**Figure 1:**
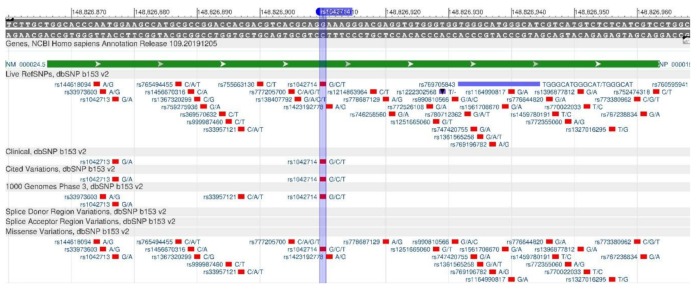
A portion of the ADRB2 gene (located on the long (q) arm of chromosome 5) containing the oligonucleotide polymorphism rs1042714.

**Figure 2:**
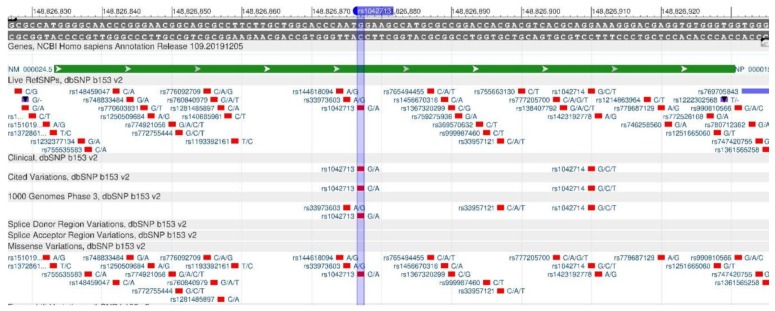
A portion of the ADRB2 gene (located on the long (q) arm of chromosome 5) containing the oligonucleotide polymorphism rs1042713.

ADRB2 encodes the β2-adrenergic receptor involved in the regulation of cardiac, pulmonary, vascular, endocrine and central nervous systems [15]. There are rs1042714 (Figure 1) and rs1042713 (Figure 2) polymorphisms in the gene, which causes a decrease in the rate of carbohydrate output in cells, and, therefore, leads the development of type 2 diabetes mellitus, obesity and metabolic syndrome [16].

**Figure 3:**
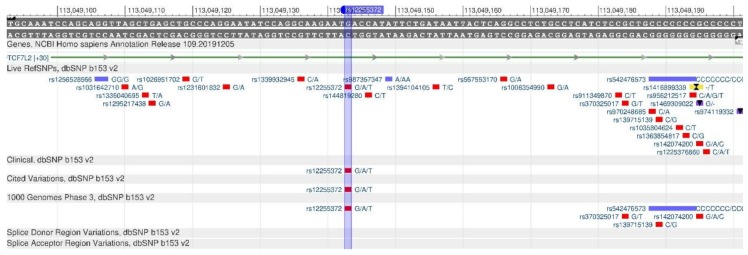
The TCF7L2 gene region (located on the long (q) arm of chromosome 10) containing the oligonucleotide polymorphism rs12255372.

**Figure 4:**
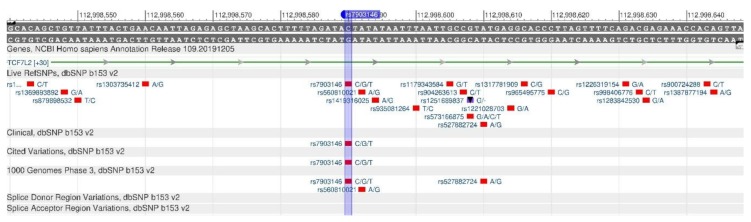
**The** TCF7L2 gene region (located on the long (q) arm of chromosome 10) containing the oligonucleotide polymorphism rs7903146.

TCF7L2 encodes a protein that acts as a transcription factor and participates in the formation of pancreatic β-cells producing insulin needed to reduce blood sugar. There are rs12255372 (Figure 3) and rs7903146 (Figure 4) polymorphisms, when there is insufficient insulin in the body, as its production is disturbed, resulting in a high risk of type 2 diabetes mellitus [17,18]. 

**Figure 5:**
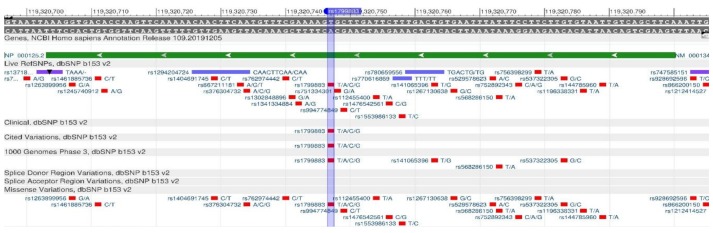
A plot of the FABP2 gene (located on the long (q) arm of chromosome 4) containing the oligonucleotide polymorphism rs1799883.

The FABP2 gene encodes a protein that binds fatty acids in the intestines and promotes their active transportation through the intestinal wall membrane [19]. The rs1799883 polymorphism (Figure 5) leads to the fact that the fat-binding protein acquires a greater affinity to fatty acids when the body assimilates fats from food more efficiently, and, therefore, there is a high risk of increased body mass index (BMI), obesity and type 2 diabetes mellitus [20].

**Figure 6:**
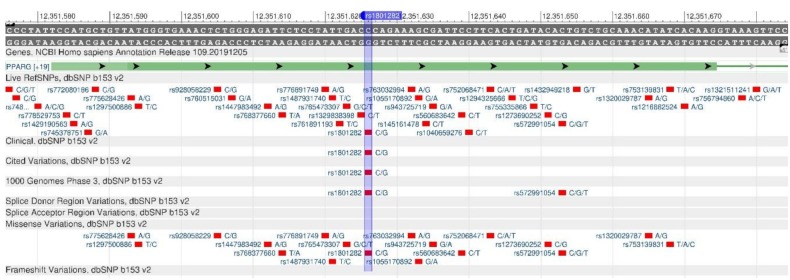
A portion of the PPARG gene (located on the short (p) arm of chromosome 3) containing the oligonucleotide polymorphism rs1801282.

The PPARG gene encodes the nuclear receptor (gamma receptor), which induces the proliferation of peroxisomes regulating the transcription of various genes involved in the metabolism of lipids and carbohydrates—in muscle tissues metabolism and in the flammatory processes of human body [21]. Researchers believe that rs1801282 (Figure 6) causes increased sensitivity to insulin, total cholesterol, HDL (high-density lipoprotein) and increased glucose utilization, which serves as a protective mechanism against diabetes mellitus and obesity [22].

**Figure 7:**
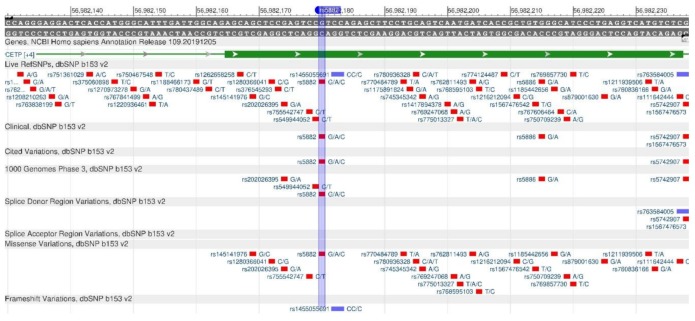
A portion of the CETP gene (located on the long (q) arm of chromosome 16) containing the oligonucleotide polymorphism rs5882.

The CETP gene is one of the key lipid metabolism genes, as it encodes the carrier protein of cholesterol esters, i.e., the gene translates “good” HDL cholesterol into “bad” LDL (low density lipoprotein) [23]. Rs5882 (Figure 7) is a cause of change in the primary structure of the protein, resulting in a decrease in HDL and increased LDL, i.e., in atherosclerosis and ischemic diseases of the heart and vessels [24].

**Figure 8:**
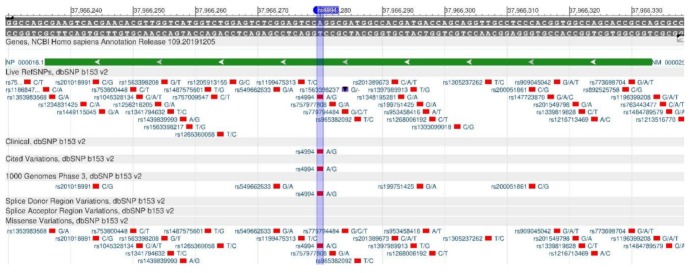
A region of the ADRB3 gene (located on the short (p) arm of chromosome 8) containing the oligonucleotide polymorphism rs4994.

The ADRB3 gene encodes the protein involved in the lipolysis regulation. Rs4994 polymorphism (Figure 8) reduces the sensitivity of protein, changing its structure, due to which there is a slowdown of oxidation and increased fat accumulation, causing obesity [25,26].

**Figure 9:**
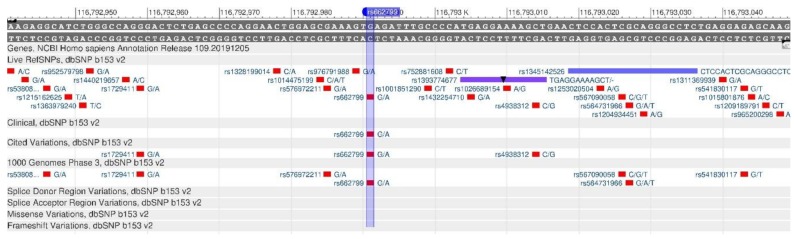
ApoA5 gene region (located on the long (q) arm of chromosome 11) containing the oligonucleotide polymorphism rs662799.

**Figure 10:**
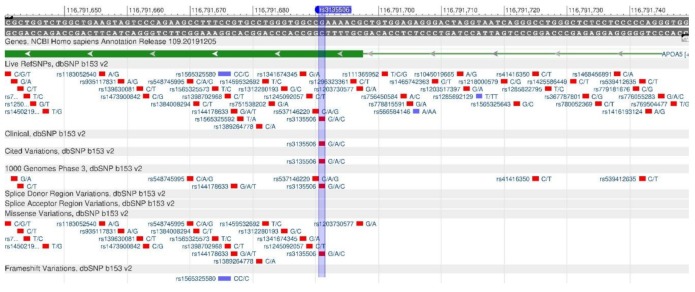
ApoA5 gene region (located on the long (q) arm of chromosome 11) containing the oligonucleotide polymorphism rs3135506.

Apolipoprotein A5 is the protein that is encoded by the APOA5 gene. It plays an important role in regulating the level of triglycerides in blood plasma [27]. The gene polymorphisms are associated with a change in the triglyceride level. In the case of rs662799 and rs3135506 oligonucleotide mutations (Figure 9 and Figure 10), there is a decrease in the amount of apolipoprotein A5 and, as a result, there is an increase in the level of triglycerides and VLDL in human blood. Therefore, the risk of atherosclerosis and cardiovascular diseases increases [28].

**Figure 11:**
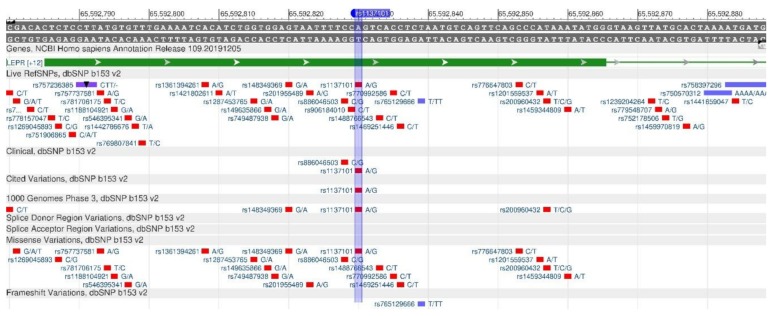
A portion of the LEPR gene (located on the short (p) arm of chromosome 1) containing the oligonucleotide polymorphism rs1137101.

The LEPR gene encodes the protein (leptin receptor) that is sensitive to insulin. It participates in the body weight regulation and energy metabolism [29]. A decreased receptor production, which is observed in rs1137101 polymorphism (Figure 11), causes leptin resistance and, as a result of which, fats in cells increase, and, therefore, obesity is developing. Moreover, lack of leptin leads to increased appetite, as there is no feeling of saturation [30]. There are forms of monogenic obesity caused by a mutation of LEPR gene [31].

**Figure 12:**
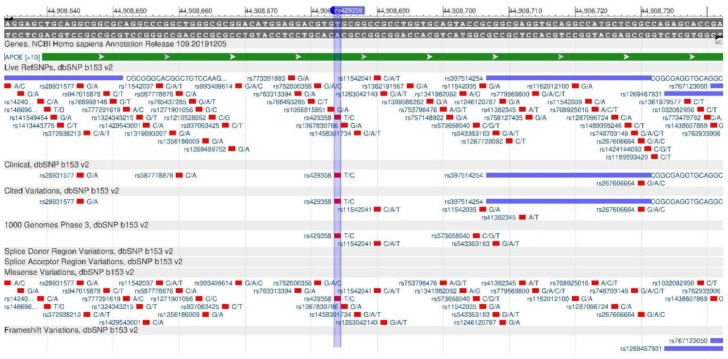
ApoE gene region (located on the long (q) arm of chromosome 19) containing the oligonucleotide polymorphism rs429358.

**Figure 13:**
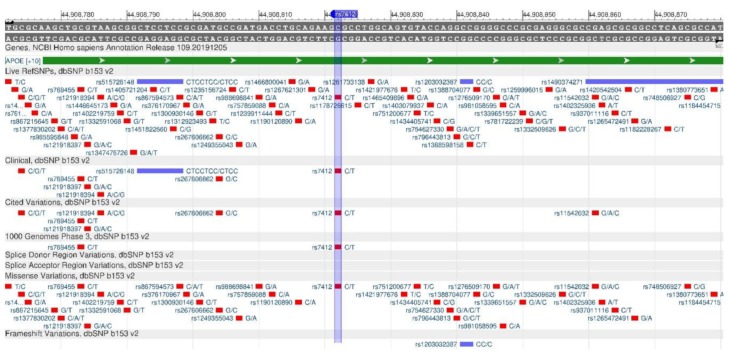
ApoE gene region (located on the long (q) arm of chromosome 19) containing the oligonucleotide polymorphism rs7412.

The ApoE gene encodes the apolipoprotein E protein, which provides the absorption of cholesterol through B- and E-receptors and promotes the absorption of VLDLP (very low-density lipoprotein) by the liver. The missense mutations rs429358 (Figure 12) and rs7412 (Figure 13) affect the cholesterol metabolism and cause a deterioration of the cholesterol removal process. In the case of the ApoE3/E3 genotype, the risk of atherosclerosis is minimal, while, in the case of the ApoE4/E4 genotype, it is the highest [32,33]. 

### 2.2. Genes Associated with Food Intolerances 

The list includes HLA-DQ and MCM6 (rs4988235) genes which cause monogenic diseases. 

The HLA genes are part of the immune-response mechanism, i.e., they help the immune system to distinguish the body’s own proteins from foreign ones—viruses and bacteria. HLA-DQ genes are responsible for the immunological recognition of cells. The produced proteins of HLA-DQ2 and HLA-DQ8 genes form a functional protein complex—the antigen-binding dimer (DQαβ), when affixing to peptides outside the cell, recognizes them as foreign or own proteins, triggering an immune response in the first case [34,35]. These genes can cause an autoimmune disease in the case of inadequate immune response: for example, an inadequate response to gluten proteins, which cause the inflammation that damages body organs and tissues and lead to the signs and symptoms of celiac disease. The predisposition to the disease is observed in the presence of HLA-DQ2 and HLA-DQ8 genes [36].

**Figure 14:**
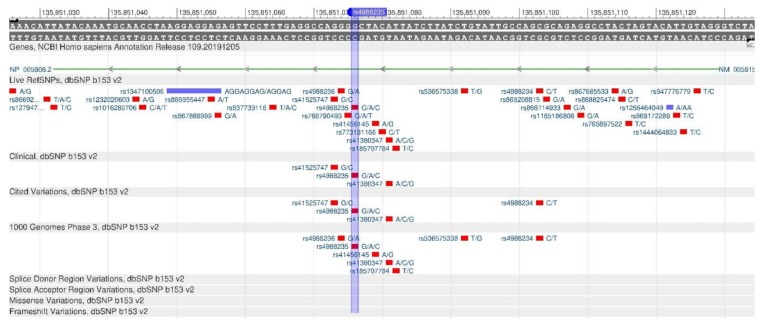
A portion of the MCM6 gene (located on the long (q) arm of chromosome 2) containing the oligonucleotide polymorphism rs4988235.

The MCM6 gene helps control the expression of a nearby LCT gene, which encodes lactose protein, an enzyme capable of digesting lactose contained in milk and dairy products [37]. The rs4988235 polymorphism in the LCT and MCM6 genes leads to the ability of digesting milk in adulthood [38]. The gene sequence is shown in Figure 14. 

We also considered the dynamics of dominant and recessive allele frequencies in the populations for this gene (Table 2).

### 2.3. Genes Responsible for the Metabolism of Vitamins 

The genes responsible for the metabolism of vitamins are: BCMO1 (rs7501331, rs12934922, rs119478057), ALPL (rs1256335) and NBPF3 (rs4654748), MTNFR (rs1801133), FUT2 (rs602662), VDR (rs1544410) and GC (rs2282679), F17ADS1 (rs1 4547). For this list of genes, we considered the dynamics of genetic composition of populations, namely, dominant and recessive allele frequencies (Table 3).

**Figure 15:**
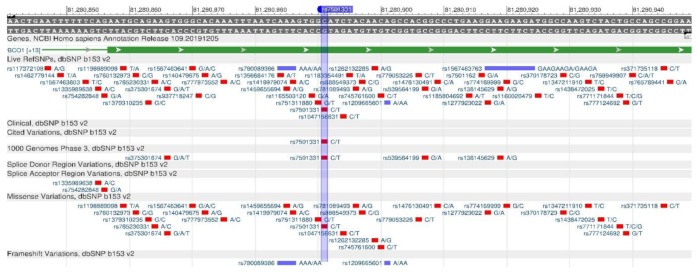
The BCMO1 gene region (located on the long (q) arm of chromosome 16) containing the oligonucleotide polymorphism rs7501331.

**Figure 16:**
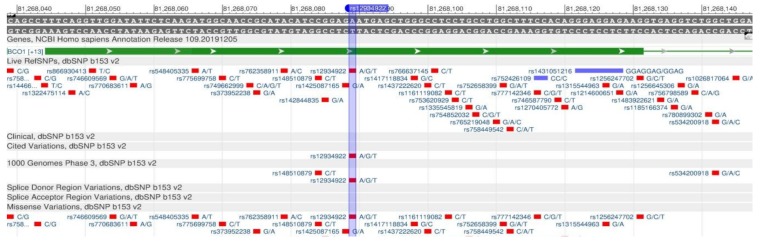
The BCMO1 gene region (located on the long (q) arm of chromosome 16) containing the oligonucleotide polymorphism rs12934922.

**Figure 17:**
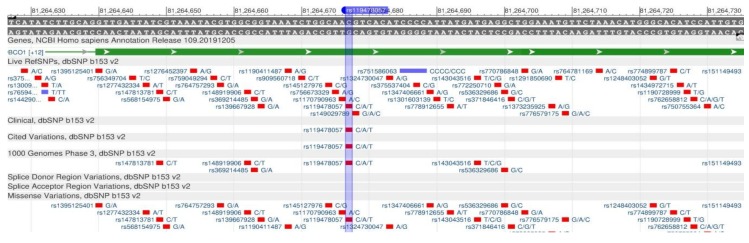
The BCMO1 gene region (located on the long (q) arm of chromosome 16) containing the oligonucleotide polymorphism rs119478057.

The BCMO1 gene encodes β-Carotene Oxygenaze1, which is a key enzyme in the breakdown of beta-carotene to vitamin A. Vitamin A is important for the body, as it is one of the main nutrients involved in the lipid metabolism regulation, control of adipocyte differentiation and lipid tissue exchange [50]. Rs7501331 (Figure 15), rs12934922 (Figure 16) and rs119478057 (Figure 17) cause a decrease in the enzyme synthesis rate, which leads to a deterioration in the vitamin A digestibility [51].

**Figure 18:**
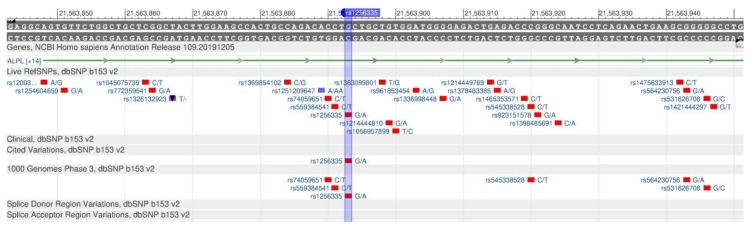
The ALPL gene region (located on the short (p) arm of chromosome 1) containing the oligonucleotide polymorphism rs1256335.

**Figure 19:**
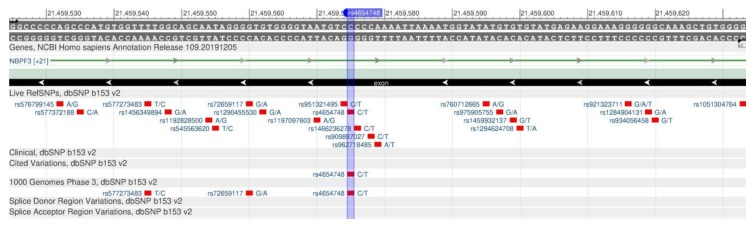
The NBPF3 gene region (located on the short (p) arm of chromosome 1) containing the oligonucleotide polymorphism rs4654748.

The ALPL and NBPF3 genes enzyme belongs to the hydrolase group—alkaline phosphatase—necessary for the association with the synthesis of neurotransmitters in the central nervous system (CNS) [52]. One of such neurotransmitters is gamma-aminobutyric acid (GABA). Vitamin B6 affects its synthesis. In other words, the alkaline phosphatase deficiency leads to the vitamin B6 deficiency in the central nervous system, and, therefore, causes neurological defects, as vitamin B6 is important for the brain development and functioning [53]. The vitamin B6 deficiency is observed in the case of rs1256335 (ALPL, Figure 18) and rs4654748 (NBPF3, Figure 19) polymorphisms.

**Figure 20:**
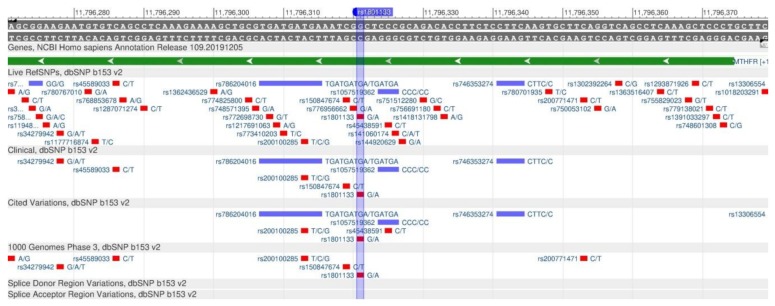
A portion of the MTHFR gene (located on the short (p) arm of chromosome 1) containing the oligonucleotide polymorphism rs1801133.

The MTNFR gene encodes the methylenetetrahydrofolate reductase protein, which participates in the metabolism of folic acid, necessary for converting homocysteine to methionine and further into S-adenosylmethionine, which plays an important role in the DNA methylation process [54]. As a result of the rs1801133 polymorphism (Figure 20), the metabolic pathway of homocysteine transformation is disturbed and its content in plasma increases. High homocysteine levels increase the probability of atherosclerosis, thrombosis and type 2 diabetes mellitus [55].

**Figure 21:**
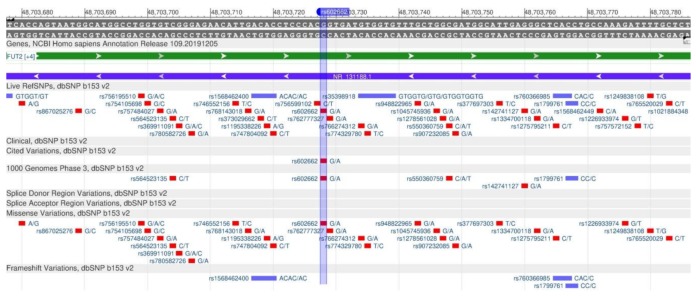
A portion of the FUT2 gene (located on the long (q) arm of chromosome 19) containing the oligonucleotide polymorphism rs602662.

The FUT2 gene encodes protein fucosyltransferase 2, which is involved in the synthesis of Lewis blood group antigens. These antigens contribute to the attachment of gastric pathogens to the stomach mucous membrane which can affect the vitamin B12 absorption [56]. The vitamin B12 deficiency, observed with a poor vitamin absorption in the intestine, is associated with anemia, cardiovascular diseases, cancer and neurodegenerative disorders. The rs602662 polymorphism (Figure 21) reduces the risk of impaired vitamin B12 absorption in the intestine; therefore, there is an increase in B12 in the blood [57].

**Figure 22:**
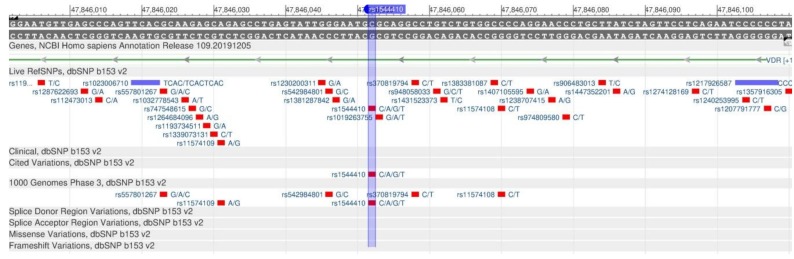
A section of the VDR gene (located on the long (q) arm of chromosome 12) containing the oligonucleotide polymorphism rs1544410.

**Figure 23:**
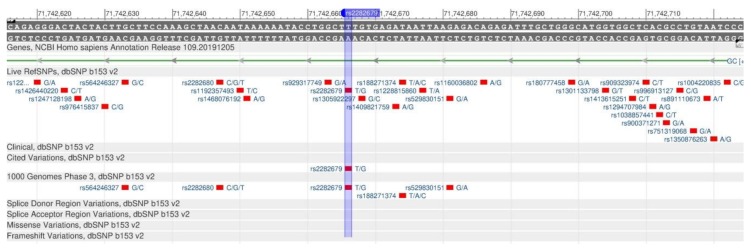
A portion of the GC gene (located on the short (p) arm of chromosome 4) containing the oligonucleotide polymorphism rs2282679.

The active form of vitamin D inhibits the development of breast, colon and prostate cancer, has a positive effect on the cardiovascular system and prevents autoimmune diseases [58]. Two genes are responsible for the vitamin D metabolism: VDR and GC. The VDR gene synthesizes the protein, vitamin D receptor, which participates in the metabolism of calcium and phosphates essential for bones and teeth. The rs1544410 polymorphism (Figure 22) reduces receptor sensitivity to vitamin D, and, therefore, there is an increase in the calcium removal from bones. As a result, there is a decrease in the mineral bone density, and the risk of osteoporosis increases [59]. The GC gene encodes the vitamin D binding protein; its rs2282679 polymorphism (Figure 23) is associated with a change in the level of vitamin D in the blood [60].

**Figure 24:**
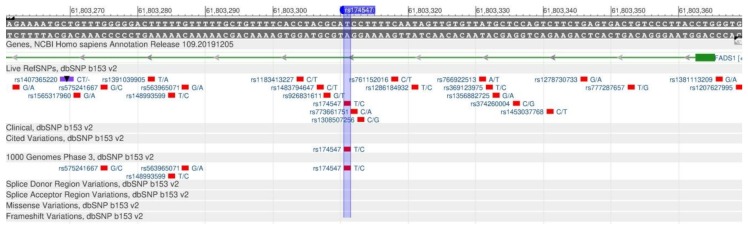
The FADS1 gene region (located on the long (q) arm of chromosome 9) containing the oligonucleotide polymorphism rs174547.

The FADS1 gene synthesizes the fatty acid desaturase 1 protein (FADS), which is able to synthesize important polyunsaturated fatty acids (eicosapentaenoic and arachidonic acids) from omega-3 substrates and omega-6, respectively. In other words, the gene controls the metabolism of fatty acids in the body [61,62]. The rs174547 polymorphism (Figure 24) is associated with a decrease in the level of omega-3 fatty acids, an increase in the relative level of omega-6 fatty acids and the concentration of transunsaturated fatty acids, resulting in the development of coronary heart disease, type II diabetes mellitus, metabolic syndrome and obesity [63].

### 2.4. Genes Responsible for Taste Sensations

Taste plays an important role in the assessment of the nutritional composition of the food consumed. GLUT2 (rs5400) is responsible for sweet sensitivity, TAS2R38 (rs1726866)—for bitter taste, CD36 (rs1761667) is associated with the taste sensitivity to and preference for fat. ADD1 (rs4961) and CYP11B2 (rs1799998) are associated with salt sensitivity. The gene sequences containing oligonucleotide polymorphisms are shown in Figure 4. For this list of genes, we considered the dynamics of genetic composition of populations, namely dominant and recessive allele frequencies (Table 4).

**Figure 25:**
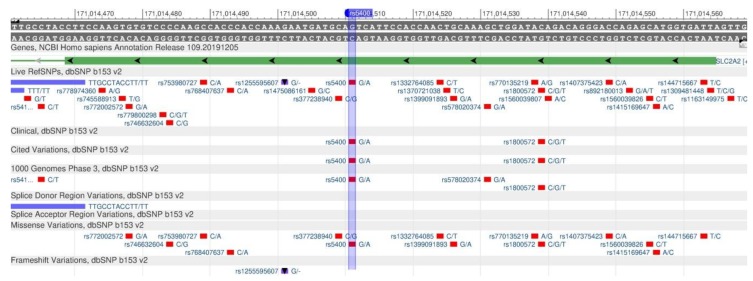
The GLUT2 gene region (located on the long (q) arm of chromosome 3) containing the rs5400 oligonucleotide polymorphism.

The GLUT2 (or SLC2A2) gene encodes the protein that transports glucose through the cell membrane, resulting in the gene being a “sensor” of glucose sensitivity [69]. In the case of the rs5400 polymorphism (Figure 25), there is a decrease in sugar sensitivity and, therefore, it leads to its excessive consumption resulting in a high risk of type 2 diabetes [70,71]. 

**Figure 26:**
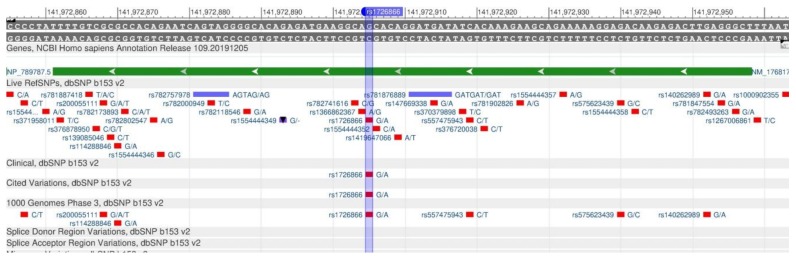
The TAS2R38 gene region (located on the long (q) arm of chromosome 7) containing the oligonucleotide polymorphism rs1726866.

The TAS2R38 gene encodes the protein of tongue cells, which controls the ability to feel glucosinolates, a family of bitter compounds [72]. In other words, this gene is associated with a hypersensitivity to bitter tastes. In the case of the rs1726866 polymorphism (Figure 26), there is a decrease in bitter susceptibility and, therefore, such people can consume bitter foods rich in antioxidants. The sensitivity to bitter taste can affect both diet and taste preferences, and metabolic hormonal regulation [73]. 

**Figure 27:**
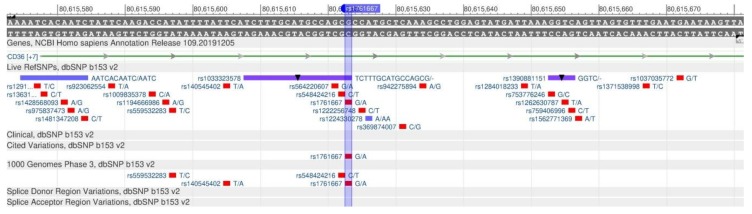
A plot of the CD36 gene (located on the long (q) arm of chromosome 7) containing the oligonucleotide polymorphism rs1761667.

The CD36 gene encodes the protein that participates in the fat recognition in food and their absorption in the intestine [74]. The rs1761667 (Figure 27) polymorphism in this gene is associated with a disturbance in the perception of fatty acids and, therefore, there is an increase in their consumption. As a result of polymorphism, there is a risk of diabetes mellitus and metabolic syndrome development [75]. 

**Figure 28:**
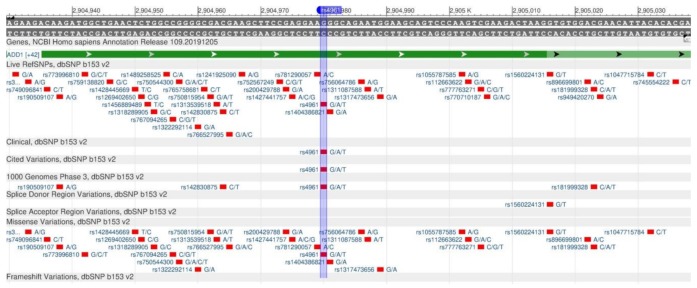
A portion of the ADD1 gene (located on the short (p) arm of chromosome 4) containing the oligonucleotide polymorphism rs4961.

The ADD1 gene encodes the structural protein of the cell (α-adducin), which is involved in the transport of sodium ions through kidneys [76]. The rs4961 polymorphism (Figure 28) is associated with the disruption of sodium ion transport and salt-sensitive hypertension [77,78]. The protein with impaired function cannot effectively remove salt from the body and it leads to the water-salt imbalance, edema and high blood pressure, essential hypertension and cardiovascular diseases [79]. 

**Figure 29:**
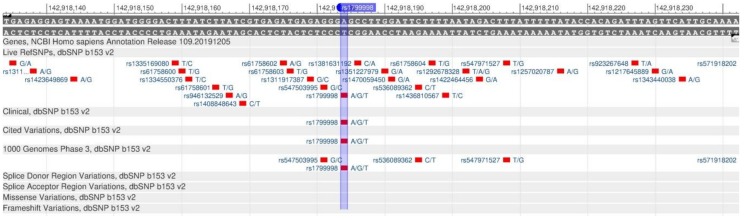
A plot of the CYP11B2 gene (located on the long (q) arm of chromosome 8) containing the oligonucleotide polymorphism rs1799998.

The CYP11B2 gene encodes the protein that participates in the synthesis of the aldosterone hormone [80]. Aldosterone regulates blood pressure, increasing it. It also maintains salt and fluid levels in the cells. In the case of the rs1799998 polymorphism (Figure 29), there is an increase in the rate of aldosterone synthesis, resulting in a fluid retention, body swelling and high blood pressure [81]. 

### 2.5. Genes Responsible for the Metabolism of Xenobiotics

**Figure 30:**
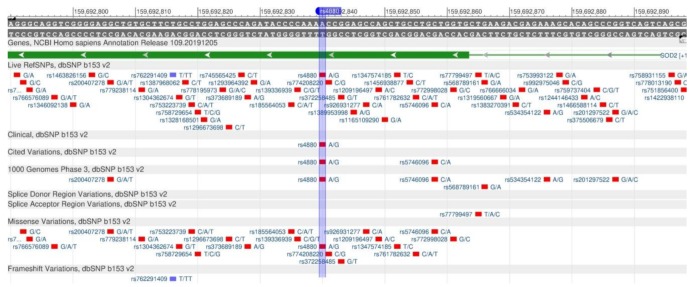
A portion of the MnSOD gene (located on the long (q) arm of chromosome 6) containing the oligonucleotide polymorphism rs4880.

MnSOD (rs4880), GSTP1 (rs947894) and CYP1A2 (rs762551) participate in the oxidation of xenobiotics entering the body with food. The MnSOD gene (also known as SOD2) codes superoxide dismutase, which binds to the by-products of oxidative phosphorylation and converts them into hydrogen peroxide and diatomic oxygen. In other words, the enzyme destroys xenobiotics which are toxic for the body. The rs4880 polymorphism (Figure 30) is associated with a decreased enzyme activity, an increased cellular damage and an increased risk of diseases associated with a DNA damage, e.g., cardiovascular diseases and malignant tumors [82,83]. 

**Figure 31:**
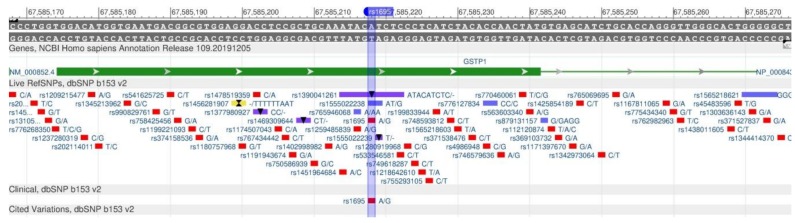
The GSTP1 gene region (located on the long (q) arm of chromosome 11) containing the oligonucleotide polymorphism rs947894.

GSTP1 encodes the protein (glutathione S-transferasep-1), which detoxifies xenobiotics by their joining glutathione contained in erythrocytes [84]. Rs947894 (Figure 31) leads to a decrease in the enzyme activity and, consequently, to an increased sensitivity to carcinogens and toxins, and their increased accumulation in the body. 

**Figure 32:**
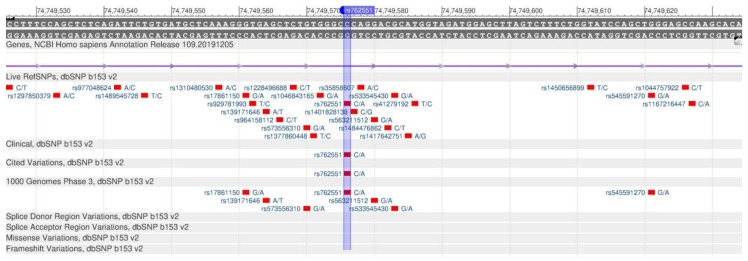
A plot of the CYP1A2 gene (located on the long (q) arm of chromosome 15) containing the oligonucleotide polymorphism rs762551.

CYP1A2 encodes the enzyme of cytochrome P450 system, which plays an important role in the oxidation of endogenous and exogenous compounds [85]. This gene participates in the metabolism of caffeine, food mutagens and medicines. The slower the metabolism is, the longer the xenobiotic circulates in the blood, and the more damage it causes to the body [86]. In the case of the rs762551 polymorphism (Figure 32), there is an increase in the xenobiotics metabolism rate [87]. 

For this list of genes, we considered the dynamics of genetic composition of populations, namely, dominant and recessive allele frequencies (Table 5).

### 2.6. Genes Responsible for Eating Preferences

The list of genes influencing eating preferences includes: FTO (rs9939609), MC4R (rs17782313), DRD2 (rs1800497). In this study, eating preferences means the tendency to overeat caused by genetic polymorphisms.

**Figure 33:**
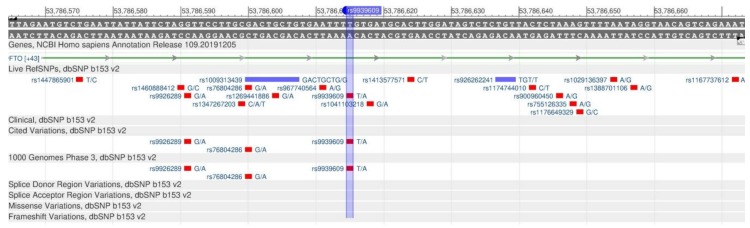
A portion of the FTO gene (located on the long (q) arm of chromosome 16) containing the oligonucleotide polymorphism rs9939609.

The FTO gene encodes the protein that participates in energy metabolism, oxidative reactions and the metabolism of fatty acids. rs9939609 (Figure 33) is associated with an increase in the body mass index (BMI) and obesity [91,92]. The excessive expression of FTO is associated with a higher food intake and subsequent increases in body weight and fat, assuming that the level of energy expenditure and physical activity remain unchanged [93].

**Figure 34:**
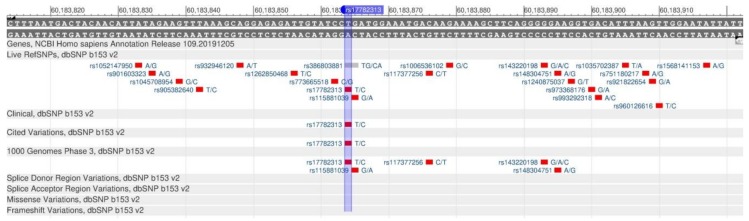
A portion of the MC4R gene (located on the long (q) arm of chromosome 18) containing the oligonucleotide polymorphism rs17782313.

The MC4R gene encodes the protein, which is a membrane-bound receptor that plays an important role in energy homeostasis and eating preferences regulation. The protein, in association with melantropin, becomes responsible for the feeling of saturation [94]. In addition, the MC4R gene plays a key role in the regulation of glucose homeostasis [95]. The 17782313 polymorphism (Figure 34) is the cause of autosomal-dominant obesity, as the gene is associated with BMI, eating preferences and regulation of food consumption [96]. 

**Figure 35:**
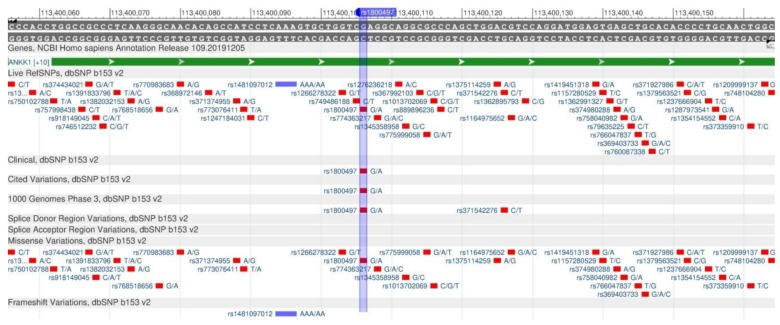
A plot of the DRD2 gene (located on the long (q) arm of chromosome 11) containing the oligonucleotide polymorphism rs1800497.

DRD2 encodes the dopamine receptor (D2), which inhibits the adenyl cyclase activity. Dopamine is responsible for many processes occurring in the central nervous system: eating, addiction to alcohol, smoking and drugs [97]. The expression of DRD2 gene is affected by the near-by ANKK1 gene, which the rs1800497 polymorphism (known as Taq1A, Figure 35) leads to a decrease in the dopamine production, and, as a result, the body begins to look for the ways of increasing the hormone of joy by addictions [98]. 

The dynamics of the genetic composition of populations, namely the frequency of dominant and recessive alleles for these genes, are presented in Table 6.

### 2.7. Genes Responsible for Food Addiction

Genes responsible for the development of food addiction include: ADH1B (rs1229984) and ALDH2 (rs671), CHRNA5 (rs16969968) and CHRNA3 (rs1051730).

**Figure 36:**
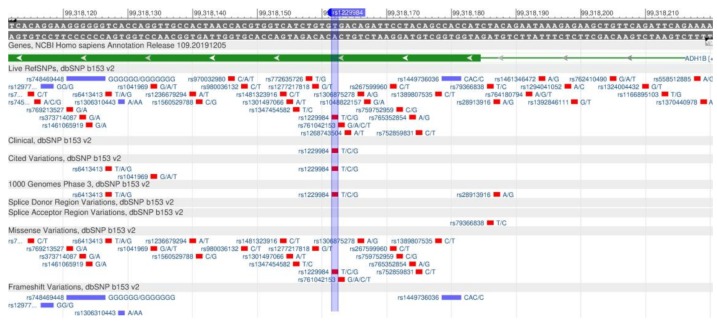
A portion of the ADH1B gene (located on the long (q) arm of chromosome 4) containing the oligonucleotide polymorphism rs1229984.

The ADH1B and ALDH2 genes are responsible for the sensitivity to alcohol [102]. The ADH1B gene encodes protein, which is part of the family of alcoholic dehydrogenase (beta-subunit), oxidizing ethanol, retinol and other aliphatic alcohols, and lipid peroxidation products. The rs1229984 polymorphism (Figure 36) leads to an increase in the enzyme activity, and, therefore, to the increased rate of ethanol decay, thereby removing it from the blood. 

**Figure 37:**
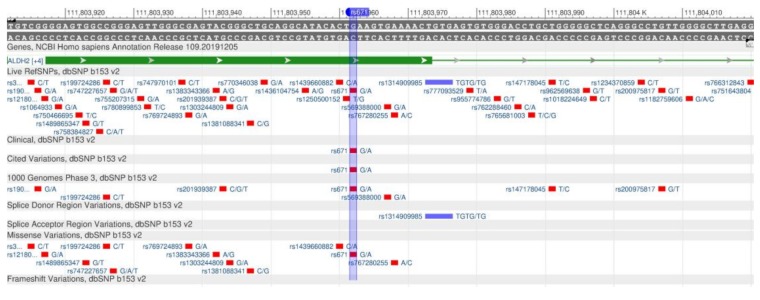
A section of the ALDH2 gene (located on the long (q) arm of chromosome 12) containing the oligonucleotide polymorphism rs671.

The rapid ethanol decay to acetaldehyde leads to severe hangover syndrome, as acetaldehyde circulates in the blood for a longer time and causes unpleasant symptoms. As a result, alcoholism is unlikely to occur [103]. The ALDH2 gene encodes the enzyme aldehyde dehydrogenase, which participates in the acetaldehyde oxidation to acetate. In the case of the rs671 polymorphism (Figure 37), the aldehyde dehydrogenase enzyme loses its activity. Researchers suggest that the inactivity is the cause of alcohol intolerance in Asian and Northern peoples [104].

**Figure 38:**
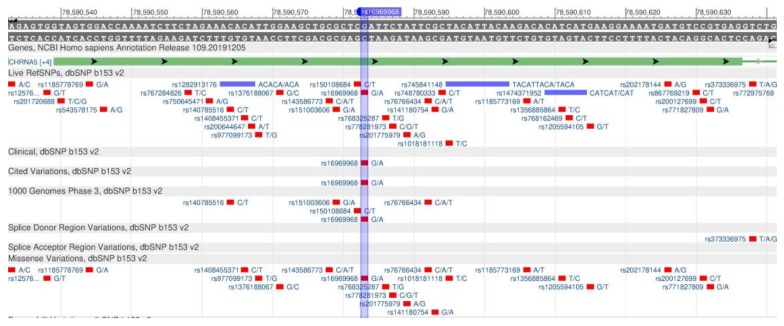
A portion of the CHRNA5 gene (located on the long (q) arm of chromosome 15) containing the oligonucleotide polymorphism rs16969968.

**Figure 39:**
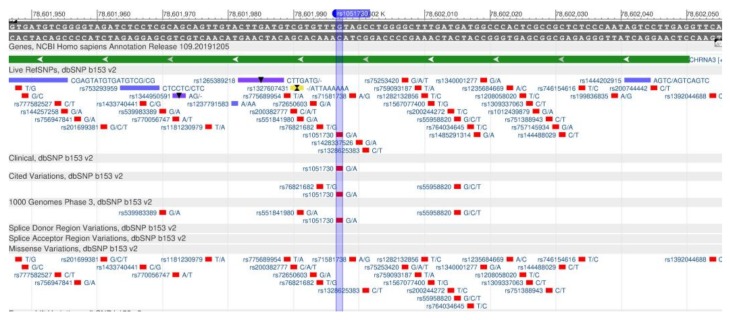
A region of the CHRNA3 gene (located on the long (q) arm of chromosome 15) containing the oligonucleotide polymorphism rs1051730.

The CHRNA5 and CHRNA3 genes encode the proteins (receptors α-5 and α-3, respectively), which are subunits of the nicotine acetylcholine receptor. The genes increase the nicotine dependence and provoke smoking-related diseases [105]. The rs16969968 polymorphisms in CHRNA5 (Figure 38) [106] and CHRNA3 rs1051730 (Figure 39) are associated with the increased nicotine dependence and risk of lung cancer [107,108]. 

The dynamics of the genetic composition of populations, namely, the frequency of dominant and recessive alleles for these genes, are presented in Table 7.

As a result of the review, we compiled a list of 34 genes (Table 8), divided into seven categories depending on their function in metabolism. In Table 8, also the gene localization on chromosomes and genotype are given. 

Table 8 presents 34 genes: nine genes responsible for metabolism of carbohydrates and fats, two genes for food intolerance, eight genes for metabolism of vitamins, five genes for taste sensations, three genes for metabolism of xenobiotics, seven genes for eating preferences. In Table 8, polymorphism of some genes is not indicated, e.g., for HLA-DQ genes, since the development of celiac disease depends not on genes polymorphisms, but on their presence. For the ApoE gene, two polymorphisms rs429358 and rs7412 are indicated, which form the three main variants of the gene (ApoE-ε2, ApoE-ε3 and ApoE-ε4), and, so, there are six possible combinations of the ApoE gene: E2/2, E2/3, E3/3, E4/2, E4/3, E4/4, which are presented in Table 8. Two names are presented for the genotype of DRD2, CYP1A2, ADH1B and ALDH2 genes.

#### Achievements of Nutrigenetics for Personalized Diet Development

At present, there are widely available programs, in which individual data (weight, height, and age) can be filled in and, afterwards, the appropriate physical activity and diet are selected. The programs can include not only height and weight data, but also the information on diseases, allergic reactions, blood test indicators, and, on this base, the individual diet is specified, e.g., Metabolic balance German app [126,127]. 

Today, one can find databases that combine information for the research in the field of nutrigenomics, e.g., Oxford scientists NutriGenomeDB [128,129], and it allows entering the gene or genes of interest and get information about their expression. The data can be obtained in Excel and PDF formats which are convenient for a further use. The information about the initial experiment (nutriet, additional data about the experiment, etc.) is also displayed. That is, the idea of NutriGenomeDB is to quantify the similarity of gene expression with biologically active food components. Due to the data, it is possible to develop new functional products.

Recently, a model of a personalized diet has been developed, and it includes individual restrictions (past medical history, DNA, habitat, climate, life style and energy expenditure) and the purpose of the diet (to maintain health or physical fitness, longevity, taste preferences, for a balanced diet that promotes fast saturation with a small portion) [130]. It is based on:

1. Information architecture, i.e., protected databases, where the information about human genes and other personal data are collected.
2. Service technology, a website or mobile app, where the questionnaires are placed; a program that analyzes all the data and gives general recommendations.
3. Production technology (biologically active additives, functional products [131], new flavors, etc.).

Furthermore, a method for the formation of personalized nutrition based on DNA analysis with an emphasis on overweight and food intolerance was developed [132]. The method includes a study of the polymorphic sites of LCT, PPARG, ADRB2, FABP2, TCF7L2 genes and the identification of HLA-DQ haplotype. Depending on how the polymorphism affects the excess weight and/or food intolerance, and/or the presence of HLA-DQ haplotype, a diet is recommended. For example:

1. If the causes of overweight are associated with the polymorphism in TCF7L2 and FABP2 genes, then a 6-month diet with the limitation of saturated fats and prevention of type 2 diabetes is prescribed.
2. If the causes of overweight are associated with the polymorphism in TCF7L2 and PPARG genes, then a 6-month diet preventive of type 2 diabetes with hunger days is prescribed.
3. If the causes of overweight are associated with the polymorphism in TCF7L2 FABP2 and PPARG genes, then a 6-month low-fat diet with hunger days and preventive of type 2 diabetes is prescribed.
4. If the causes of overweight are associated with the polymorphism in CF7L2 and ADRB2 genes, then a 6-month low-carbohydrate diet preventive of type 2 diabetes is prescribed.
5. If the causes of overweight are associated with the polymorphism in TCF7L2 ADRB2 and PPARG genes, then a 6-month low-carbohydrate diet with hunger days and preventive of type 2 diabetes is prescribed. The list can be continued.

The presented method is efficiently used by our domestic colleagues to select an individual diet. The authors believe that the method is well suited within the genes in question (LCT, PPARG, ADRB2, FABP2, TCF7L2, HLA-DQ), but these are not the only genes that can affect overweight and food intolerance. So, as part of our review, it was found that, in addition to the genes presented, the accumulation of excess mass is also influenced by the genes ADRB3, LEPR, FTO, MC4R. In addition to gluten and lactose intolerance, there is a large amount of food irritants or "allergens," e.g., albumin, biogenic amines (histamine, tiramine), sulfites, sodium glutamate, various food dyes, preservatives and sweeteners, etc. [133]. Simply for these stimuli, the gene and its polymorphisms have not been studied or little studied. In other words, the method gives good dietary recommendations, but considers a limited number of genes.

The articles proving the efficiency of the method proposed by our domestic colleagues were not found. However, in many articles, each of the listed genes takes part in overweight recruitment. Thus, the possible impact of the LCT gene on the excess body weight, cancer development, cardiovascular diseases, bone health and lipid metabolism were considered [134]. In Ref. [135], the results are presented that a high-fat/low-fat diet affects PPARG gene expression, and that, if there is polymorphism in the gene, a decrease in body weight appears. The authors of Ref. [136] used a nutrigenetic test to optimize nutrients in the human diet. They carried out genetic testing (one of the gene analyses was PPARG) and modified the Mediterranean diet to the personal requirements of the body according to the results of the study. As a result of the experiment, the weight loss in test group was more intense than that in the control group of people eating without nutrigenetic correction. Thus, nutrigenetics is a tool to improve and optimize healthy full nutrition, and it is an effective mean for long-term lifestyle change.

Genes involved in excess weight accumulation occupy an important sector in nutrigenetic research. However, in order to create a personalized diet, it is necessary to consider all the genes that affect eating behavior. Taking into account only one gene, it can lead to a deterioration of health and well-being. For example, if the study does not consider the genes responsible for food intolerance, an allergic reaction may occur. If you do not consider the genes responsible for the vitamin metabolism and assign a diet that is not rich in these biologically active substances, then beriberi will eventually develop with time, and it can lead to a decrease in immunity and the development of chronic disease [137]. If the genes responsible for eating behavior are not accounted, then there is a high risk of making an incorrect/unsuitable diet, and, as a result, an accumulation of excess weight and moral dissatisfaction with the diet. The remaining genes, which, in this review, were assigned to the groups responsible for the metabolism of xenobiotics, food habituation and taste sensations [138], must be considered for the purpose of assigning a correct recommendation to limit/eliminate/add a particular food to the daily diet. For example, unintentional exclusion from the diet of a product rich in antioxidants, in the presence of low activity of enzymes that block the free radical action in the body, can lead to the body poisoning, or become one of the factors in the cancer development.

## 3. Conclusions

### 3.1. Genes Responsible for Eating Preferences

The available data are contradictory in some respects, and a final answer about the role of genes and their polymorphisms in eating preferences and some diseases cannot be given. This is induced by the fact that the research involves a small number of people from different ethnic backgrounds and living conditions and it leads to contrasting results. However, despite this, many genes from the list can be used in DNA testing to develop nutritional strategies. The review is limited, as we used the data from published articles rather than from the original data provided by authors. There are obvious contradictions of the information presented in various databases: alleles of polymorphisms in their nucleotide expression do not coincide, and it can be seen in some figures and tables.

The results show that not all polymorphisms have a negative impact on health, e.g., due to the polymorphism in the MCM6 gene, people in their adulthood can consume milk and dairy products. From the data obtained, it can be said that the number of polymorphisms causing monogenic diseases is inferior to the number of genes leading to polygenic diseases. The role of genes is extensive, and, in addition to their influence on eating preferences, they (their mutations) can be among the causes of the following diseases:

- obesity (ADRB2, FABP2, PPARG, ADRB3, LEPR, FTO, MC4R);
- type 2 diabetes (ADRB2, TCF7L2, FABP2, PPARG, CETP, GLUT2, CD36);
- cardiovascular diseases (CETP, ApoA5, ApoE, ADD1, CYP11B2, MnSOD);
- cancer (MnSOD, GSTP1, CYP1A2, CHRNA5, CHRNA3);
- metabolic syndrome (ADRB2, TAS2R38, CD36).

The study of population genetics, allele frequencies of the genes shows that:

1) For the African (AFR) population, it is not advisable to consider the following list of genes having a frequency of recessive allele occurrence less than 20%: ADRB2 (rs1042714), PPARG (rs1801282), ADRB3 (rs4994), ApoA5 (rs662799, rs3135506), ApoE (rs7412), MCM6 (rs4988235), BCMO1 (rs7501331, rs12934922, rs119478057), NBPF3 (rs4654748), MTNFR (rs1801133), GC (rs2282679), FADS1 (rs174547), ADD1 (rs4961), CYP11B2 (rs1799998), ADH1B (rs1229984), ALDH2 (rs671), CHRNA5 (rs16969968), CHRNA3 (rs1051730).
2) For the American (AMR) population, it is not advisable to consider the following list of genes having a frequency of recessive allele occurrence less than 20%: PPARG (rs1801282), ADRB3 (rs4994), ApoA5 (rs662799, rs3135506), ApoE (rs429358, rs7412), BCMO1 (rs7501331, rs119478057), ALPL (rs1256335), GLUT2 (rs5400), ADD1 (rs4961), MC4R (rs17782313), ADH1B (rs1229984), ALDH2 (rs671).
3) For the East Asian population (EAS), a list of polymorphisms has been identified, and their effect in metabolism cannot be considered: TCF7L2 with the rs12255372 polymorphism, since the dominant allele has 90% frequency, and with the rs7903146 polymorphism, where the dominant allele has the 98% frequency; ApoA5 gene with the rs3135506 mutation, since the dominant allele occurrence frequency is 100%, the LEPR gene rs1137101, since the dominant allele frequency is 87%; the MCM6 rs4988235, since the dominant allele frequency is 100%. Furthermore, BCMO1 (rs119478057), ALPL (rs1256335), FUT2 (rs602662), VDR (rs1544410), MnSOD (rs4880), GSTP1 rs947894 (rs1695) are inappropriate to consider, since the recessive allele occurrence frequency is less than 20%.
4) For the European population (EUR), a list of polymorphisms has been identified, and their effect on the metabolism cannot be considered: PPARG (rs1801282), ADRB3 (rs4994), ApoA5 (rs662799, rs3135506), ApoE (rs429358, rs7412), BCMO1 (rs119478057), GLUT2 (rs5400), DRD2 (rs1800497), ADH91B (rs122929291B 84), ALDH2 (rs671).
5) For the South Asian population (SAS), a list of polymorphisms has been identified, and their effect on the metabolism cannot be considered: PPARG (rs1801282), ADRB3 (rs4994), ApoA5 (rs662799, rs3135506), ApoE (rs429358, rs7412), MCM6 (rs4988235), BCMO1 (rs119478057), MTNFR (rs1801133), FADS1 (rs1 (rs1801133) 174547), GLUT2 (rs5400), ADH1B (rs1229984), ALDH2 (rs671), CHRNA5 (rs16969968), CHRNA3 (rs1051730).
6) For the six genes, it turns out to be inappropriate to consider how their oligonucleotide mutations affect eating preferences, as the allele occurrence dynamics is either minimal or absent. So, for PPARG rs1801282, the occurrence of allele G for all populations is 7%, and, therefore, the search for the recessive allele polymorphism will yield a negative result. The list also includes: ADRB3 (rs4994, recessive allele occurrence frequency 12%), ApoA5 (rs3135506, recessive allele occurrence frequency 6%), ApoE (rs7412, recessive allele occurrence frequency 8%), BCMO1 (rs119478057, recessive allele occurrence frequency 0%), ALDH2 (rs671, recessive allele occurrence frequency 4%).
7) Regarding the previous conclusion, the list of genes, in which the mutations play a role in polygenic diseases, changes:
  - obesity (ADRB2, FABP2, LEPR, FTO, MC4R);
  - type 2 diabetes (ADRB2, TCF7L2, FABP2, PPARG, CETP, GLUT2, CD36);
  - cardiovascular diseases (CETP, ADD1, CYP11B2, MnSOD);
  - cancer (MnSOD, GSTP1, CYP1A2, CHRNA5, CHRNA3);
  - metabolic syndrome (ADRB2, TAS2R38, CD36).

Taking into account population genetics data is important, as it will allow excluding false positive results about the disturbed/normal metabolism, i.e., the consumer will not need to spend money to determine polymorphisms in the gene, which it cannot initially have. For a recessive allele to be of interest in the study of a gene polymorphism responsible for eating preferences, it is necessary that its frequency be no less than 20%.

### 3.2. Methods and Programs for Developing Personal Eating Plans

The weak points of available methods and apps for developing personalized diets are:

1) they do not consider genetic data;
2) the diets are difficult to follow (both in the choice of products and in the mode), therefore, a consumer often has to quit, and it is harmful for a body;
3) applications are not translated into an appropriate language;
4) they are mostly to be paid for;
5) the information on genetic predispositions is difficult to understand;
6) population genetics are not considered.

**Figure 40:**
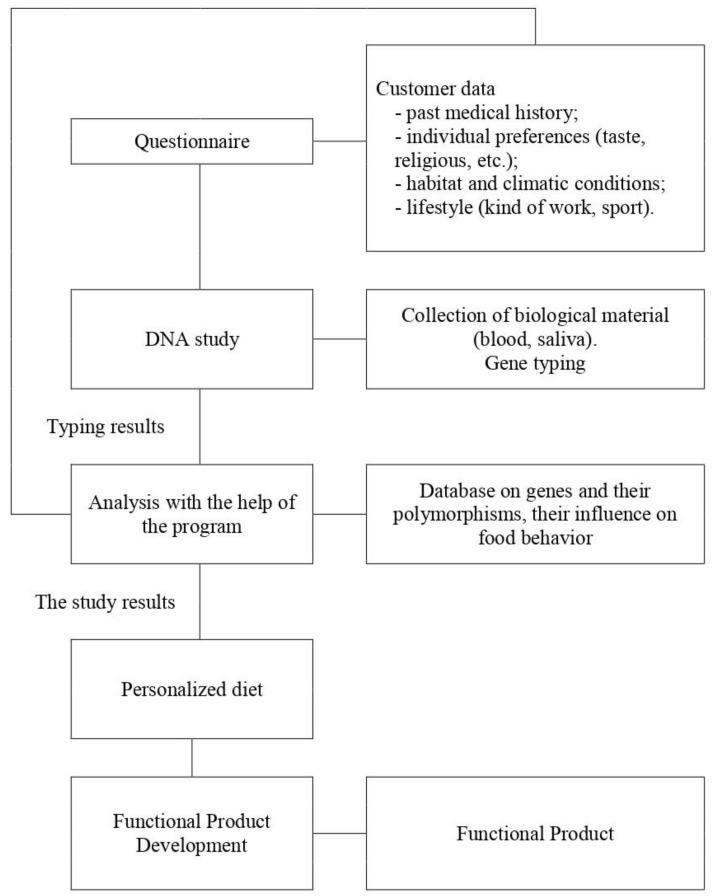
The model for a personalized diet, the ultimate goal of which is the production of a functional product.

We designed an example of the model suitable for developing personalized diets (Figure 40). 

The model consists of five blocks, including:

1) a questionnaire, where past medical history anamnesis, individual preferences (taste and religious ones), habitat and climatic zone of residence, and lifestyle are specified. All data must be filled in a secure database which will later be used for the analysis of DNA results;
2) DNA study. Depending on the purpose of a diet (to prevent obesity, diabetes, to improve well-being, etc.), a gene or a set of genes is chosen, taking into account the population genetics data. The material is collected (venous blood or saliva, buccal epithelium), DNA is extracted and the necessary polymorphisms are determined;
3) data analysis. The program analyzes all the data and produces the result;
4) developing nutritional strategies. An expert in nutrigenetics has all the data and develops a personal eating plan based on a genetic make-up.
5) developing a functional product. With a customer permission, the selection of optimal foods, nutrients and biologically active substances is made to develop a functional product on the basis of the genetic make-up and psycho-emotional preferences of a customer [139].

### 3.3. General Conclusions

To make the nutrigenetic research more popular, more detailed and reliable, it is necessary to:

1. change the way of thinking, for both doctors and consumers, regarding dieting, since today one approach to all health and diet problems is prevalent, and it can only aggravate the body condition;
2. create conditions for the cooperation of sciences (genetics, nutritiology, dietary science);
3. make up genetics databases containing unified, clear and detailed information, for customers and prospective scientists;
4. train new nutrigenetic specialists interested in carrying out research work and capable of giving competent interpretation results;
5. establish a legal framework in the field of nutrigenetic research, including developing DNA-based diets;
6. design a model for individual diets, based on a patient’s genetic makeup.

One of the goals of nutrigenetics is to provide personalized diets which are very promising in disease prevention. Nevertheless, personalized nutrition depends not only on the genetics, but also on the psycho-emotional needs of a person.

## Figures and Tables

**Table 1:** Population genetics for genes responsible for absorption of carbohydrates and fats.

Gene	Polymorphism	Alleli	Frequency of Occurrence in Populations						Reference
			all, %	AFR, %	AMR, %	EAS, %	EUR, %	SAS, %	
ADRB2	rs1042714	G:	20	14	24	7	41	55	[2]
		C:	80	86	76	93	59	45	
	rs1042713	G:	52	48	54	45	61	55	[3]
		A:	48	52	46	55	39	45	
TCF7L2	rs12255372	G:	79	70	78	99	71	78	[4]
		T:	21	30	22	1	29	22	
	rs7903146	C:	77	74	77	98	68	70	[5]
		T:	23	26	23	2	32	30	
FABP2	rs1799883	T:	25	22	23	25	27	31	[6]
		C:	75	78	77	75	73	69	
PPARG	rs1801282	C:	93	99	88	97	88	88	[7]
		G:	7	1	12	3	12	12	
CETP	rs5882	G:	47	64	40	44	33	45	[8]
		A:	53	36	60	56	67	55	
ADRB3	rs4994	A:	88	91	88	87	92	84	[9]
		G:	12	9	12	12	8	16	
ApoA5	rs662799	G:	16	12	15	29	8	19	[10]
		A:	84	88	85	71	92	81	
	rs3135506	G:	94	93	88	100	93	96	[11]
		C:	6	7	12	0	7	4	
LEPR	rs1137101	A:	42	41	56	13	53	50	[12]
		G:	58	59	44	87	47	50	
ApoE	rs429358	T:	85	73	90	91	85	91	[13]
		C:	15	27	10	9	15	9	
	rs7412	C:	92	90	95	90	94	96	[14]
		T:	8	10	5	10	6	4	

Abbreviations mean: AFR—African; AMR—American; EAS—East Asian; EUR—European; SAS—South Asian.

**Table 2:** Population genetics of genes responsible for lactose intolerance.

Gene	Polymorphism	Alleli	Frequency of Occurrence in Populations						Reference
			all, %	AFR, %	AMR, %	EAS, %	EUR, %	SAS, %	
MCM6	rs4988235	G:	84	97	78	100	49	89	[39]
		A:	16	3	22	0	51	11	

Abbreviations mean: AFR—African; AMR—American; EAS—East Asian; EUR—European; SAS—South Asian.

**Table 3:** Population genetics of genes responsible for vitamin metabolism.

Gene	Polymorphism	Alleli	Frequency of Occurrence in Populations						Reference
			all, %	AFR, %	AMR, %	EAS, %	EUR, %	SAS, %	
BCMO1	rs7501331	C:	85	99	83	81	77	79	[40]
		T:	15	1	17	19	23	21	
	rs12934922	A:	77	91	68	87	56	77	[41]
		T:	23	9	32	13	44	23	
	rs119478057	C	100	100	100	100	100	100	[42]
		T	0	0	0	0	0	0	
ALPL	rs1256335	G:	17	24	14	2	22	21	[43]
		A:	83	76	86	98	78	79	
NBPF3	rs4654748	C:	62	94	57	42	53	56	[44]
		T:	38	6	43	58	47	44	
MTNFR	rs1801133	G:	75	91	53	70	64	88	[45]
		A:	24	9	47	30	36	12	
FUT2	rs602662	G:	67	51	65	100	53	72	[46]
		A:	33	49	35	0	47	28	
VDR	rs1544410	C:	70	73	74	94	60	52	[47]
		T:	30	27	26	6	40	48	
GC	rs2282679	T:	80	95	79	74	75	70	[48]
		G:	20	5	21	26	25	30	
FADS1	rs174547	T:	70	98	41	43	65	86	[49]
		C:	30	2	59	57	35	14	

Abbreviations mean: AFR—African; AMR—American; EAS—East Asian; EUR—European; SAS—South Asian.

**Table 4:** Population genetics of genes responsible for taste sensations.

Gene	Polymorphism	Alleli	Frequency of Occurrence in Populations						Reference
			all, %	AFR, %	AMR, %	EAS, %	EUR, %	SAS, %	
GLUT2	rs5400	G	78	51	83	98	86	84	[64]
		A	22	49	17	2	14	16	
TAS2R38	rs1726866	G:	57	67	71	68	46	36	[65]
		A:	43	33	29	32	54	64	
CD36	rs1761667	G:	61	65	47	69	47	71	[66]
		A:	39	35	53	31	53	29	
ADD1	rs4961	G:	79	95	83	55	80	80	[67]
		T:	21	5	17	45	20	20	
CYP11B2	rs1799998	A:	65	81	53	71	51	61	[68]
		G:	35	19	19	29	49	39	

In the Table 4, the abbreviations mean: AFR—African; AMR—American; EAS—East Asian; EUR—European—European; SAS—South Asian.

**Table 5:** Population genetics of genes responsible for metabolism of xenobiotics.

Gene	Polymorphism	Alleli	Frequency of occurrence in populations						Reference
			all, %	AFR, %	AMR, %	EAS, %	EUR, %	SAS, %	
MnSOD	rs4880	A:	59	58	42	88	53	49	[88]
		G:	41	42	58	12	47	51	
GSTP1	rs947894(rs1695)	A:	65	52	52	82	67	71	[89]
		G:	35	48	48	18	33	29	
CYP1A2	rs762551	C:	37	44	24	33	32	47	[90]
		A:	63	56	76	67	68	53	

In the table, abbreviations mean: AFR—African; AMR—American; EAS—East Asian; EUR—European; SAS—South Asian.

**Table 6:** Population genetics of genes responsible for eating preferences.

Gene	Polymorphism	Alleli	Frequency of Occurrence in Populations						Reference
			all, %	AFR, %	AMR, %	EAS, %	EUR, %	SAS, %	
FTO	rs9939609	T:	66	51	74	83	59	71	[99]
		A:	34	49	26	17	41	29	
MC4R	rs17782313	T:	76	72	87	81	76	68	[100]
		C:	24	28	13	19	24	32	
DRD2	rs1800497	G:	67	61	69	59	81	69	[101]
		A:	33	39	31	41	19	31	

In the table, abbreviations mean: AFR—African; AMR—American; EAS—East Asian; EUR—European; SAS—South Asian.

**Table 7:** Populational genetics of genes responsible for food addiction.

Gene	Polymorphism	Alleli	Frequency of Occurrence in Populations						Reference
			all, %	AFR, %	AMR, %	EAS, %	EUR, %	SAS, %	
ADH1B	rs1229984	T:	16	0	6	70	3	2	[109]
		C:	84	100	94	30	97	98	
ALDH2	rs671	G:	96	100	100	83	100	100	[110]
		A:	4	0	0	17	0	0	
CHRNA5	rs16969968	G:	85	98	79	97	63	82	[111]
		A:	15	2	21	3	37	18	
CHRNA3	rs1051730	G:	83	91	78	97	63	82	[112]
		A:	17	9	22	3	37	18	

In Table 7, the abbreviations mean: AFR—African; AMR—American; EAS—East Asian; EUR—European; SAS—South Asian.

**Table 8:** List of genes and polymorphisms responsible for eating preferences.

Function	Reference	Gene	Polymorphism	Localization	Genotype					
					norm/norm		norm /mut		mut/mut	
Fats and carbohydrates absorption	[2]	ADRB2	rs1042714	5q32.	C/C		C/G		G/G	
	[3]		rs1042713		G/G		G/A		A/A	
	[4]	TCF7L2	rs12255372	10Q25.3	G/G		G/T		T/T	
	[5]		rs7903146		C/C		C/T		T/T	
	[113]	FABP2	rs1799883	4q26	G/G		G/A		A/A	
	[7]	PPARG	rs1801282	3p25.2	C/C		C/G		G/G	
	[8]	CETP	rs5882	16q13	G/G		G/A		A/A	
	[114]	ADRB3	rs4994	8p11.23	T/T		T/C		C/C	
	[10]	ApoA5	rs662799	11q23.3	A/A		A/G		G/G	
	[11]		rs3135506		C/C		G/C		G/G	
	[12]	LEPR	rs1137101	1p31.3	A/A		A/G		G/G	
	[13]	ApoE	rs429358	19q13.32	E2/2	E2/3	E3/3	E4/2	E4/3	E4/4
					T/T	T/T	T/T	C/T	C/T	C/C
	[14]		rs7412		T/T	C/T	C/C	C/T	C/C	C/C
Food intolerances	[36]	HLA-DQ	HLA-DQA1 HLA-DQB1	6p21.3	HLADQ2HLADQ8					
	[115]	MCM6	rs4988235	2q21.3	C/C			C/T		T/T
Metabolism of vitamins	[40]	BCMO1	rs7501331	16q23.2	C/C			C/T		T/T
	[41]		rs12934922		A/A			A/T		T/T
	[42]		rs119478057		C/C			C/T		T/T
	[43]	ALPL	rs1256335	1p36.12	G/G			G/A		A/A
	[44]	NBPF3	rs4654748		C/C			C/T		T/T
	[116]	MTHFR	rs1801133	1p36.22	C/C			C/T		T/T
	[46]	FUT2	rs602662	19q13.33	A/A			A/G		G/G
	[117]	VDR	rs1544410	12q13.11	A/A			A/G		G/G
	[118]	GC	rs2282679	4p12	A/A			A/C		C/C
	[49]	FADS1	rs174547	9q31.3	C/C			C/T		T/T
Taste sensations	[119]	GLUT2	rs5400	3q26.2	C/C			C/T		T/T
	[120]	TAS2R38	rs1726866	7q34	C/C			C/T		T/T
	[66]	CD36	rs1761667	7q21.11	G/G			G/A		A/A
	[67]	ADD1	rs4961	4p16.3	G/G			G/T		T/T
	[121]	CYP11B2	rs1799998	8q24. 3	C/C			C/T		T/T
Metabolism of xenobiotics	[122]	MnSOD	rs4880	6q25.3	C/C			C/T		T/T
	[89]	GSTP1	rs947894(rs1695)	11q13.2	A/A			A/G		G/G
	[90]	CYP1A2	rs762551	15q24.1	C/C orCYP1A2*1C			C/A		A/A or CYP1A2*1F
Eating preferences	[99]	FTO	rs9939609	16q12.2	T/T			T/A		A/A
	[100]	MC4R	rs17782313	18q21.32	T/T			T/C		C/C
	[123]	DRD2	rs1800497	11q23.2	C/C or A2/A2			C/T or A2/A1		T/T or A1/A1
Food addiction	[124]	ADH1B	rs1229984	4q23	A/A or *1/*1			A/G or*1/*2		G/G*2/*2
	[110]	ALDH2	rs671	12q24.12	G/G or *1/*1			G/A or*1/*2		A/A or*2/*2
	[111]	CHRNA5	rs16969968	15q25.1	A/A			A/G		G/G
	[125]	CHRNA3	rs1051730		C/C			C/T		T/T
